# Maternity care during COVID-19: a protocol for a qualitative evidence synthesis of women’s and maternity care providers’ views and experiences.

**DOI:** 10.12688/hrbopenres.13233.1

**Published:** 2021-02-18

**Authors:** Valerie Smith, Sarah-Jane Flaherty, Karen Matvienko-Sikar, Hannah Delaney

**Affiliations:** 1 School of Nursing & Midwifery, University of Dublin, Trinity College, Dublin, D02 T283., Ireland; 2 School of Public Health, University College Cork, Cork, Ireland

**Keywords:** COVID-19, maternity care, pregnancy and childbirth, systematic review, thematic synthesis, qualitative evidence synthesis

## Abstract

**Background:** Considerable changes in maternity care provision internationally were implemented in response to COVID-19. Such changes, often occurring suddenly with little advance warning, have had the potential to affect women’s and maternity care providers experience of maternity care, both positively and negatively. For this reason, to gain insight and understanding of personal and professional experiences, we will perform a synthesis of the available qualitative evidence on women and maternity care providers’ views and experiences of maternity care during COVID-19.

**Methods and analysis**: A qualitative evidence synthesis will be conducted. Studies will be eligible if they include pregnant or postpartum women (up to six months) and maternity care providers who received or provided care during COVID-19. To retrieve relevant literature the electronic databases of CINAHL, EMBASE, MEDLINE, PsycINFO, and the Cochrane COVID study register ( https://covid-19.cochrane.org/) will be searched from 01-Jan-2020 to date of search. A combination of search terms based on COVID-19, pregnancy, childbirth and maternity care, and study design, will be used to guide the search.  The methodological quality of the included studies will be assessed by at least two reviewers using the Evidence for Policy and Practice Information (EPPI)-Centre 12-criteria quality assessment tool. The Thomas and Harden approach to thematic synthesis will be used for data synthesis. This will involve line by line coding of extracted data, establishing descriptive themes, and determining analytical themes. Confidence in the findings of the review will be assessed by two reviewers independently using Grading of Recommendations Assessment, Development and Evaluation-Confidence in the Evidence from Reviews of Qualitative research (GRADE-CERQual).

**Conclusion**: The proposed synthesis of evidence will help identify maternity care needs during a global pandemic from the perspectives of those receiving and providing care. The evidence will inform and help enhance care provision into the future.

## Introduction

As of 20 January 2021, approx. 1-year since the emergence of coronavirus disease 2019 (COVID-19), the disease caused by the strain of coronavirus, severe acute respiratory syndrome coronavirus 2 (SARS-CoV-2), almost 97 million people have become infected globally and over two million people have died from the disease ^1^. COVID-19 affects infected individuals in varying ways, although the elderly and those with underlying co-morbidities appear more vulnerable to severe adverse outcomes ^2^. The risk of contracting COVID-19 does not appear heightened by pregnancy, nor are pregnant women more likely to die from the disease; however there is some evidence to suggest that morbidity may be higher with COVID-19 in pregnancy ^3, 4^. For example, in a living systematic review of risk and outcome data related to COVID-19, pregnant women with COVID-19, when compared to non-pregnant women of reproductive age, were more likely to need admission to intensive care (Odds Ratio (OR) 1.62, 95% Confidence Interval (CI) 1.33-1.96) and invasive ventilation (OR 1.88, 95% CI 1.36-2.60; 4 studies, 91,606 women). In a study comparing COVID positive and negative pregnant women, an increased risk of preterm birth (OR 3.34, 95% CI 1.60–7.00) and caesarean section (OR 3.63, 95% CI 1.95–6.76) were identified in COVID positive pregnant women ^5^. Results across studies are conflicting, however, and other studies exploring risks and outcomes have found no or minimal differences between COVID positive and negative pregnant groups ^4, 6^.

Irrespective of clinical risk and outcomes, as with many healthcare areas, considerable changes in the provision of maternity care internationally were implemented in response to the pandemic. Such changes, often occurring suddenly with little advance warning ^7^ and, in some instances, arguably countering the core tenets of respectful maternity care ^8^, continue to remain in place or have been lifted and reinstated as second and subsequent waves of increased virus transmission occurred. Changes to maternity care provision and practices include, but are not limited to, polices of restrictive visiting and access (e.g. partners not permitted to attend labour and birth; one designated parent for babies in neonatal intensive care units; no visiting in antenatal, postnatal and gynaecology wards), reconfiguration of physical space to accommodate suspected or confirmed COVID-19 positive women, and diversion of hospital outreach or community services back to the main hospital setting. Women’s choice for place of birth during the pandemic has also been reduced in some countries. In the UK, for example, approx. one third of NHS Trusts suspended home birthing services ^9^. This presents an interesting conundrum considering that pregnant women may have heightened concerns about exposing themselves and their babies to the virus in a hospital environment, potentially resulting in an increased demand for homebirth services at this time ^10^. Suspensions of key services such as parent education, antenatal classes and birth reflection clinics have also occurred as a result of the pandemic and antenatal and postnatal telephone or online consultations (telehealth) have increased. Throughout the pandemic, and especially as subsequent waves of increased COVID-19 transmission occur internationally, the numbers of health or maternity care professionals available to provide care has also been affected, with many absent from work as a result of infection, or self-isolating due to close contact with confirmed cases ^11^.

The changes to maternity care provision in response to COVID-19 are likely well intended. Central to their implementation is minimising the risk of COVID-19 transmission in pregnant women and maternity care providers. However, these changes have the potential to impact both positively or negatively on women’s experiences of maternity services, and on the experiences of maternity care providers in providing care to women and their families also. For instance, restrictions on partner attendance at antenatal visits, and during the birthing process can reduce women’s sense of support during pregnancy and labour ^12^. Conversely, restricted visiting postpartum can provide women with the space to bond with their babies or to establish breastfeeding, for example, and may create increased space for maternity care providers to spend time with women antenatally and postpartum. Restrictions on partner attendance, however, may also reduce opportunities for prenatal parental bonding due to missing important prenatal milestones and check-ups. Furthermore, the rapidity with which some changes occurred (almost overnight in some instances as countries entered lockdown phases), and the variation in these changes between and within countries may have led to confusion, uncertainty, and anxiety as women felt unprepared and uninformed of the services available to them, the processes involved, and the possible risk to themselves and their baby ^12^. The increased use of telehealth also poses a challenge, in particular, for individuals with poor technological literacy and/or language difficulties, potentially contributing to inequities in access to care ^8^.

The COVID-19 pandemic has impacted and affected maternity care across the globe. To gain insight and understanding of the experiences of women and maternity care providers, and to explore their views and perceptions of maternity care during COVID-19, we plan to conduct a systematic review and synthesis of the available qualitative evidence; a qualitative evidence synthesis (QES). In carrying out this QES we will identify care needs during a global pandemic which will inform and help optimise and enhance care provision into the future.

### Aim

To synthesise the available qualitative evidence on women’s and maternity care providers’ views and experiences of maternity care during COVID-19 (protocol).

The proposed review is registered with the international register of systematic reviews (PROSPERO: CRD42021232684, 29 ^th^ January 2021) and adheres to the PRISMA-P reporting guidelines for systematic review protocols (see reporting guidelines ^13^).

## Protocol

### Review methodology

A QES will be performed. QES methodology has been chosen as it promotes an increased understanding and insight of a phenomenon of interest by bringing together multiple perspectives, including contradictory views. A QES allows for the examination of similarities and differences across settings, and may lead to a new interpretative model or framework ^14– 16^. Findings from the proposed QES will offer maternity stakeholder derived evidence, based on similar and diverse experiences and perspectives of care during a global pandemic which may inform the development or implementation of maternity care guidelines or interventions into the future.

### Eligibility criteria

The SPIDER (Sample, Phenomenon of Interest, Design, Evaluation, and Research type) tool ^17^ was used to structure the eligibility criteria for the inclusion and exclusion of primary studies in the review. These criteria are:

- **S**ample: Pregnant or postpartum women of any parity or risk status, antenatal and up to six months postpartum. Maternity care providers; that is midwives, obstetric nurses, obstetricians and/or doctors involved in caring for pregnant and postpartum women during COVID-19. Maternity care providers may extend to other professionals (e.g. physiotherapists) directly involved in maternity care provision, as might be described in an included study.
- **P**henomenon of **i**nterest: Maternity care during COVID-19. For purposes of this review maternity care is broadly defined and may involve care within hospital, community or home birth settings, or as defined by the authors of an included study. The focus of this QES on maternity care during COVID-19 means that our sample of interest must have been recruited to/participated in a study any time onwards from 01 January 2020.
- **D**esign: All identified published and unpublished studies providing qualitative data on women’s and maternity care providers’ views and experiences of maternity care during COVID-19. This will include qualitative descriptive and exploratory studies, phenomenology, grounded theory, ethnography, and action research. Studies of mixed methods design, where qualitative data can be extracted separately, will be included. Survey designs with open-ended questions that provide qualitative data **may be considered** for inclusion; surveys that provide limited qualitative data (e.g. exemplar quotes to support quantitative ‘counts’) will be excluded, or where the qualitative data has not been subjected to a formal analytical approach (e.g. thematic analysis).
- **E**valuation of outcomes: The outcomes of interest to this review are views, experiences and perspectives. This means that included studies must provide in-depth qualitative or narrative data that are representative of women’s and maternity care providers’ views and experiences of maternity care during COVID-19. Studies that report numerical representations (e.g. thematic ‘counts’) of views or experiences will be excluded.
- **R**esearch type: Published and unpublished studies, in English language, from 01 January 2020 to present will be included. Abstracts deemed eligible may be included depending on the level of data provided, and whether these data can contribute to the synthesis in a meaningful way.

### Search strategy

To retrieve relevant literature, a systematic search of the electronic databases, limited by year from 01 January 2020 to present is planned. The following databases will be searched: CINAHL, EMBASE, MEDLINE, PsycINFO, and the Cochrane COVID study register. Searches will not be limited on language. However, as we are unable to translate non-English publications, and to avoid misrepresentations as a result of language nuance and contextual elements in attempting to translate, studies published in English only will be included. Searching all languages will allow us identify numbers of potentially eligible non-English publications, and, depending on how many we might find, whether this presents as a source of language bias. Keywords and subject terms used to guide the search are presented in Table 1, and will be adapted as appropriate across the different databases The search strings were developed based on the sample, phenomenon of interest, evaluation of outcomes and study type eligibility criteria, with search terms related to the latter two combined in a single search string.

**Table 1.** Search terms for electronic database search.

**S**	mother OR woman OR women OR midwives OR midwife* OR nurs* OR clinician OR physician OR doctor OR obstetric* OR professional
**PI**	(maternity ADJ care) OR healthcare OR ‘health-care’ OR matern* OR birth* OR childbirth OR prenan* OR labour OR labor OR antenatal OR antepartum OR postnatal OR postpartum OR post-partum OR puerperium AND coronavirus* OR corona virus* OR COVID-19 OR COVID OR Covid OR Covid2019 OR SARS-CoV* OR SARSCov* OR new CoV* OR novel CoV*
**E** and **R**	experiences OR experience OR view* OR perceptions OR perception OR voices OR narratives OR qualitative OR (mixed ADJ method) OR ‘grounded theory’ OR phenomenology OR ‘action research’

To ensure our search strategy is as comprehensive as possible we will additionally search the reference lists of included studies, grey literature websites (e.g. Open Grey), and proceedings of international maternity care conferences 2020 (e.g. Normal Labour and Birth Research Conference 2020). We will also contact maternity care researchers whom we are aware are conducting research on experiences of pregnancy and childbirth during COVID-19 for information on their study’s status, or whether unpublished data might be available for inclusion in our review.

### Study selection

All citations retrieved during the searching process will be exported to EndNote and duplicates removed. Following removal of duplicates the remaining records will be uploaded to Covidence, a software package designed for preparing systematic reviews, for screening and study selection. Records will be screened independently by two reviewers, initially by title and abstract, and then at full text level as relevant. Disagreements will be resolved by consensus or by involving a third reviewer if required. The screening and selection process, including results, will be reported using the PRISMA flowchart ^18^.

### Quality appraisal of included studies

The methodological quality of the included studies will be assessed using an appraisal tool developed by the Evidence for Policy and Practice Information and Co-ordinating (EPPI) Centre for use in a systematic review of healthy eating in children ^19^. The tool consists of 12 quality appraisal criteria (A-L) across three domains; i) quality of the study reporting, ii) reliability and validity of data collection and analysis, and iii) quality of the study methods ( Table 2). Each included study will be assessed independently by two reviewers on the extent to which each quality criterion is met. Considering that even poorly conducted and/or reported studies may provide relevant ‘views’ data, all studies, irrespective of quality will be included for data extraction and synthesis purposes.

**Table 2.** Quality appraisal criteria.

***Quality of the study reporting***	A= Aims and objectives clearly reported B= Adequately described the context of the research C= Adequately described the sample and sampling methods D= Adequately described the data collection methods E= Adequately described the data analysis methods
***There was good or some*** ***attempt to establish the***	F= Reliability of the data collection tools G= Validity of the data collection tools H= Reliability of the data analysis I= Validity of the data analysis
***Quality of the methods***	J= Used the appropriate data collection methods to allow for expression of views K= Used the appropriate methods for ensuring the analysis was grounded in the views L= Actively involved the participants in the design and conduct of the study

### Data extraction and synthesis

Data extraction will be based on the aim of the review. The following data will be extracted from each included study:

- Author (lead) and month published
- Source and type of publication (journal paper, conference proceeding, abstract, etc.)
- Aim of the study
- Description of participants and the study setting (country, health facility, etc.)
- COVID context (restrictions, lockdown, COVID-related practice changes, etc.)
- Study duration/timeframes
- Method(s) of data collection and analysis
- Findings related to women’s and providers’ views of maternity care during COVID-19

A pre-designed data extraction form will be used to extract the relevant data (extended data ^13^). We will pilot the data extraction form on two studies identified from the list of included studies and refine if necessary. Data extraction will be carried out independently by two reviewers (or pairs of reviewers) and cross-checked for consistency and accuracy.

The narrative, ‘findings’ data from the included studies will be synthesised using the thematic synthesis method as described by Thomas and Harden ^20^. Data synthesis will involve three stages; i) line by line coding of extracted text, ii) development of descriptive themes and, iii) generating analytical themes from the studies’ data. To conduct line by line coding, studies’ text including relevant participant quotes, will be extracted to Nvivo11, or similar software. Similarities and differences between codes will be identified and clustered to generate descriptive themes. Analytical themes and sub-themes will be generated through further reflection, iteration, discussion and synthesis of descriptive themes. One member of the review team will conduct the thematic synthesis with iteration, reflection and discursive team meetings following each phase; that is, a meeting will be scheduled when the descriptive themes are described. The review team will discuss, reflect on and agree that these themes collectively represent the studies’ data. A similar process will take place when the analytical themes are determined. This process will enhance rigour and transparency in synthesising the qualitative data.

### Assessment of confidence in the review findings; GRADE-CERQual

**Figure 1.**
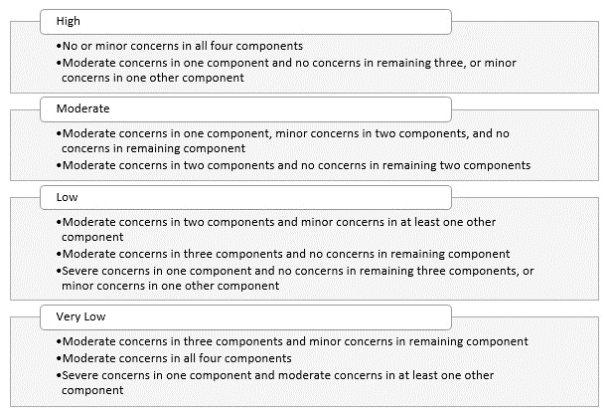
CERQual downgrading criteria.

To assess levels of confidence in the review findings, the Grading of Recommendations Assessment, Development and Evaluation-Confidence in the Evidence from Reviews of Qualitative research (GRADE-CERQual) ^21– 26^ will be applied. Using GRADE-CERQual, each discrete review finding will be assessed under four components. These are: the methodological limitations of the studies contributing to the finding, the coherence of the finding, the adequacy of data contributing to the finding and the relevance of the contributory studies to the review question. Assessments will be carried out independently by two reviewers, with final judgements based on discussions and consensus. Following these assessments, an overall assessment of confidence in each finding will be made, and categorised as High, Moderate, Low or Very Low confidence ^21^. To ensure consistency, and to provide a framework for downgrading, we have established *a priori* downgrading criteria as illustrated in Figure 1. Judgements are based on an initial assumption of ‘High confidence’ in all findings, and then downgraded accordingly.

### Dissemination of findings

The findings of this QES will be submitted for publication in an Open-Access peer-reviewed maternity-focused health journal. The findings will be shared at national and international research conferences and with identified stakeholders using dissemination methods appropriate to the stakeholder group. These will include social media posts (Facebook and Twitter), newspaper/radio media posts, and midwifery/maternity email and online forums. 

### Study status

Not yet commenced. Implementing the search strategy and screening studies for eligibility is planned for February and March 2021.

## Discussion

The findings of this QES will provide valuable insight and understanding of women’s and maternity care providers’ views and experiences of maternity care during COVID-19. This information may prove valuable for assessing how care provision may be optimised, based on the experiences of those directly involved in both receiving and providing care, as the COVID-19 pandemic continues.

## Data availability

### Underlying data

No data are associated with this article.

### Extended data

Open Science Framework: Maternity care during COVID-19; a qualitative evidence synthesis of women's and maternity care providers' views and experiences (extended files). https://doi.org/10.17605/OSF.IO/Z6DU2 ^13^

This project contains the following extended data:

- Template Data Extraction Form.docx (Template Data Extraction Form)

### Reporting guidelines

Open Science Framework: PRISMA-P checklist for ‘Maternity care during COVID-19: a protocol for a qualitative evidence synthesis of women’s and maternity care providers’ views and experiences’ https://doi.org/10.17605/OSF.IO/Z6DU2 ^13^

Data are available under the terms of the Creative Commons Attribution 4.0 International license (CC-BY 4.0).
