# Supplementary material for: Maternity care during COVID-19: a protocol for a qualitative evidence synthesis of women’s and maternity care providers’ views and experiences
Source: HRB Open Res. 2021 Feb 18;4:21. [Version 1] doi: 10.12688/hrbopenres.13233.1 (PMC8283550; doi:10.12688/hrbopenres.13233.1)
Supplement: Peer review history

# Peer review: Reviewer response for version 1

**DOI:** 10.21956/hrbopenres.14394.r29744

**Published:** 2021-07-15

**Authors:** Pauline Dawson

**Affiliations:** 1 Department of Women's and Children's Health, Dunedin School of Medicine, University of Otago, Dunedin, New Zealand

Thank you for the invitation to review this protocol. In my view, this proposal for a qualitative evidence synthesis (QES) will make a valuable contribution to the consolidation of knowledge around the experience of maternity care from the perspective of both pregnant people and carers in the context of the COVID-19 pandemic. As the authors note, maternity care was reorganised extremely swiftly in the face of the pandemic with scant evidence basis for care in such circumstances. The protocol clearly sets out a comprehensive review strategy that will inform best practice care in the future as the world continues to deal with the ongoing pandemic.

**Study Design**

 The design is well set out and provides sound detail of the frameworks and procedures the carry out the study. The COVID-19 pandemic is a recent event and ongoing so care will need to be taken to include literature published or released while the review is being carried out. I would suggest timepoints are stipulated where searches are repeated in order to be as inclusive as possible. It is encouraging to see grey literature included and an international approach taken. However, restricting articles to the English language only may cause bias although the authors have noted an approach for assessing this in the search strategy section.

 One effect of the pandemic has been a plethora of literature regarding its impact on healthcare, and there appears no shortage of qualitative literature on maternity experiences. Some of this may be due to rapid review and publishing protocols for COVID-19 related research. This factor means that a strong quality assessment protocol is required. The quality appraisal section discusses the use of the Evidence for Policy and Practice Information and Co-ordinating (EPPI) Centre tool in this process as laid out in Table 2. However, it is not clear to me how the EPPI tool quality findings will relate to synthesis when the protocol states “all studies, irrespective of quality will be included for data extraction and synthesis purposes”.

 I also have questions about the inclusion criteria of “pregnant or postpartum women” specifically. I would like to see this extended to include family/whanau/support people as these too would have been significantly impacted by changing care provision. This additional inclusion has a cultural impact specifically in societies where cultural ways of being mean that birth is a collective event centred around the birthing person. This will may also gather additional data on (in)equity and cultural disparities which have also been identified in the COVID-19 period. ^1^

 From an equity perspective, I would also suggest broadening the search terms “mother OR woman OR women” to be LBGTQ+ inclusive and so reflect all pregnant and birthing people. If these people are to be excluded, explicit justification for such should be stated.

 As this is a global review, I think more specific comment needs to be made on how international diversity will be handled in analysis and presentation. Countries (and even regions e.g. in the USA) responded differently to the onset and subsequent waves of the disease. Also, varying locations have extremes in resources and healthcare systems. Experience of a publicly funded health system compared to that in a private or insurance-based system may not be able to be directly compared. Finally, there are also diverse models of maternity care which may impact experience during the pandemic. For example, a case loading continuity midwifery model may have responded differently to a medical obstetric dominated model. How these differences are accounted for in analysis needs to be addressed

**Methods**

 The methods described are robust and utilise validated frameworks. There is enough detail to allow replication apart from, as mentioned in the review by Bohren et al. (2021) how dissent between reviewers will be resolved which needs to be expanded on.

**References**

1. Steyn N, Binny RN, Hannah K, Hendy SC, James A, Kukutai T, Lustig A, McLeod M, Plank MJ, Ridings K, Sporle A. Estimated inequities in COVID-19 infection fatality rates by ethnicity for Aotearoa New Zealand. *New Zealand Medical Journal. *2020 Sep 4;133(1521):28-39. PMID: 32994635.)

Is the study design appropriate for the research question?

Partly

Is the rationale for, and objectives of, the study clearly described?

Yes

Are sufficient details of the methods provided to allow replication by others?

Partly

Are the datasets clearly presented in a useable and accessible format?

Not applicable

Reviewer Expertise:

Maternity care, Social determinants of health, Equity

I confirm that I have read this submission and believe that I have an appropriate level of expertise to confirm that it is of an acceptable scientific standard, however I have significant reservations, as outlined above.

## References

[1] : Estimated inequities in COVID-19 infection fatality rates by ethnicity for Aotearoa New Zealand. *medRxiv* .2020; 10.1101/2020.04.20.20073437 10.1101/2020.04.20.20073437 32994635

# Peer review: Reviewer response for version 1

**DOI:** 10.21956/hrbopenres.14394.r29709

**Published:** 2021-07-15

**Authors:** Julia Sanders

**Affiliations:** 1 School of Healthcare Sciences, Cardiff University, Cardiff, UK

Thank you for the opportunity to review this timely protocol. There is increasing interest in the experiences of women of ‘socially distanced’ maternity care and the impact this has had on women and wider families, and this synthesis will generate new highly relevant information to inform practice.   

 The methods proposed are appropriate but given the anticipated limited number of surveys conducted across different countries it may be appropriate to also include published text provided in in response to open survey questions. As all published text will be a sample of that obtained in a qualitative study, and for some surveys open text replies may be detailed, the justification for excluding open text in published surveys was unclear.  The authors may wish to consider this once they have a better idea of the volume of qualitative studies currently published.

 Although the time frame for publications will be limited to the period of the pandemic, the attitudes and experiences of women may have varied considerably throughout the period. As the pandemic progressed understanding of viral transmission increased as did knowledge of the impact on women, babies, and staff. It would be important to consider the period of data collection in relation to the stage of the pandemic, knowledge levels of the virus and potential impact on pregnancy and babies.

Is the study design appropriate for the research question?

Yes

Is the rationale for, and objectives of, the study clearly described?

Yes

Are sufficient details of the methods provided to allow replication by others?

Yes

Are the datasets clearly presented in a useable and accessible format?

Not applicable

Reviewer Expertise:

Perinatal health, safety in maternity care, intervention evaluation.

I confirm that I have read this submission and believe that I have an appropriate level of expertise to confirm that it is of an acceptable scientific standard.

# Peer review: Reviewer response for version 1

**DOI:** 10.21956/hrbopenres.14394.r29405

**Published:** 2021-05-27

**Authors:** Meghan A. Bohren, Katherine Eddy

**Affiliations:** 1 Gender and Women's Health Unit, Centre for Health Equity, School of Population and Global Health, University of Melbourne, Melbourne, Australia; 2 Burnet Institute, Melbourne, Australia

Thank you for the opportunity to review this protocol. The proposed research on maternity care during COVID-19 is highly relevant and has potential to provide valuable insights for those involved in providing maternity care to women during a global pandemic. The authors have presented a clear and convincing rationale for conducting a qualitative evidence synthesis (QES) on this topic, and the proposed methods are generally sound. There are several things that we consider may strengthen the proposed research, which are outlined below.

**Study design**

 Given that the COVID-19 pandemic is relatively recent (commencing early 2020) it is likely that some relevant primary studies are not yet published. It is also possible that new studies will be conducted while this QES is ongoing. The authors should endeavour to present a comprehensive and current synthesis of available evidence. In order to do this, we suggest the authors consider updating the search at various stages of the review to bring in any newly available evidence. For example, this may be undertaken at the time of finalising the analysis, and immediately prior to publication. We also agree with the authors’ decision to include eligible studies from the grey literature. We also suggest the authors search for pre-prints which may be available on this topic, using databases including medRxiv.org.

 This review has a relatively short timeframe for eligible studies (1 January 2020-current), and we agree that this timeframe is appropriate for the topic. However, such a short timeframe may result in a limited number of eligible studies. A qualitative evidence synthesis may be undermined by either too many or too few included studies ( *ref: Booth, A. Searching for qualitative research for inclusion in systematic reviews: a structured methodological review. Syst Rev 5, 74 (2016).* *https://doi.org/10.1186/s13643-016-0249-x* ^1^). It may therefore be worthwhile considering expanding this research to a mixed-methods review, thereby including relevant quantitative evidence from surveys of women and maternity care providers. For example, if the currently available qualitative evidence is considered too thin to support a particular theme, quantitative evidence may provide additional context and support for a broader finding related to this theme. ( *ref: Noyes J, Booth A, Moore G, et al. Synthesising quantitative and qualitative evidence to inform guidelines on complex interventions: clarifying the purposes, designs and outlining some methods. BMJ Glob Health 2019;4:e000893.* *https://doi.org/10.1136/bmjgh-2018-000893* ^2^).

 In the introduction, the authors describe some of the changes that have occurred in maternity care, including restrictive visiting and access policies. These types of changes are likely to affect not only the women, but also their partners and families. The authors propose to focus the review on the views and experiences of women and maternity care providers. However, we consider the views and experiences of partners and families on these changes are likely to be connected and interact with those of the women in potentially complex ways, and that the perspectives of partners and families are relevant to the broader goal, acknowledged by the authors, of optimising and enhancing maternity care provision. We consider that including these perspectives in the review would strengthen the review findings. We therefore suggest the authors consider either expanding the scope of the review to include the views and experiences of partners and families, or provide a rationale for why these perspectives are not relevant for this QES.

 The authors state that they will conduct thematic analysis in accordance with the approach described by Thomas and Harden. However, the authors have not described how context will be accounted for in the analysis, or whether any subgroup analysis will be undertaken. While we note that some contextual factors may emerge during analysis and cannot be pre-specified, we consider there are several important phenomena that should be taken into account upfront. First, we note that the review includes both women’s and maternity care providers’ views. Even within the same setting, the perspectives and experiences of these two groups are likely to be quite different and this should be clearly accounted for in the analysis (e.g. by conducting subgroup analysis to determine which themes are relevant to each group).

 Secondly, we note that this is a global review and that experiences of COVID-19 and the policies of governments and healthcare institutions have differed significantly around the world. The authors should consider including an explanation for why a global review is nevertheless appropriate, and how this diverse experience will be accounted for in the analysis and presentation of findings.

 Thirdly, even within settings the experience of COVID-19 and the policies of governments and healthcare institutions have differed over time. For example, the experiences of women and providers during a first, second, or third wave and strong lockdown measures are likely to be quite different to women’s experiences during periods where case numbers are lower and restrictions are relaxed. We consider that it will be similarly important for the authors to take this intertemporal variation into account, and consider the localised COVID-19 context when analysing and synthesising individual study data.

**Details of methods**

 The authors have generally described the study methods in sufficient detail, notwithstanding the suggestions in relation to pre-specified subgroup analysis outlined above. We do have one minor comment in relation to resolution of differences between reviewers. The authors specify that disagreements during title and abstract screening and full text review will be resolved by consensus or by involving a third reviewer if required, and that GRADE-CERQual assessments will likewise be carried out independently with final judgements based on discussions and consensus. For clarity, we suggest the authors also specify how disagreements between reviewers will be resolved in relation to the quality appraisal of included studies and data extraction.

Is the study design appropriate for the research question?

Partly

Is the rationale for, and objectives of, the study clearly described?

Yes

Are sufficient details of the methods provided to allow replication by others?

Partly

Are the datasets clearly presented in a useable and accessible format?

Not applicable

Reviewer Expertise:

maternal health, public health, qualitative evidence synthesis

We confirm that we have read this submission and believe that we have an appropriate level of expertise to confirm that it is of an acceptable scientific standard.

## References

[1] : Searching for qualitative research for inclusion in systematic reviews: a structured methodological review. *Syst Rev* .2016;5: 10.1186/s13643-016-0249-x 74 10.1186/s13643-016-0249-x 27145932PMC4855695

[2] : Synthesising quantitative and qualitative evidence to inform guidelines on complex interventions: clarifying the purposes, designs and outlining some methods. *BMJ Glob Health* .2019;4(Suppl 1) : 10.1136/bmjgh-2018-000893 e000893 10.1136/bmjgh-2018-000893 30775016PMC6350750
